# Treatment in advanced colorectal cancer: what, when and how?

**DOI:** 10.1038/sj.bjc.6605061

**Published:** 2009-05-12

**Authors:** I Chau, D Cunningham

**Affiliations:** 1 Department of Medicine, Royal Marsden Hospital, London and Surrey, UK

**Keywords:** colorectal cancer, oxaliplatin, irinotecan, capecitabine, bevacizumab, cetuximab

## Abstract

Treatment of advanced colorectal cancer (CRC) increasingly requires a multidisciplinary approach and multiple treatment options add to the complexity of clinical decision-making. Recently novel targeted therapy against angiogenesis and epidermal growth factor receptor completed a plethora of phase III studies. The addition of bevacizumab to chemotherapy improved the efficacy over chemotherapy alone in both first and second line settings, although the magnitude of benefit may not be as great when a more optimal chemotherapy platform is used. Studies performed thus far did not address conclusively whether bevacizumab should be continued in subsequent lines of treatment. Anti-angiogenesis tyrosine kinase inhibitors have not shown any additional benefit over chemotherapy alone so far. Although some benefits were seen with cetuximab in all settings of treating advanced CRC, *K-ras* mutation status provides an important determinant of who would not benefit from such a treatment. Caution should be exercised in combining anti-angiogenesis with anti-EGFR strategy until further randomised data become available. In this review, we have focused on the implications of these trial results on the everyday management decisions of treating advanced CRC.

Treatment of advanced colorectal cancer (CRC) increasingly requires a multidisciplinary approach and multiple treatment options add to the complexity of clinical decision-making. The ability to cure some patients with metastasis confined in liver or lung has also challenged the conventional treatment approach and is now integrating both systemic treatment and locoregional approach. Recently novel targeted therapy against angiogenesis and epidermal growth factor receptor (EGFR) completed a plethora of phase III studies. In this review, we have focused on the implications of these trial results on the everyday management decisions of treating advanced CRC. Furthermore, we have discussed the duration of treatment; sequential *vs* combination treatment; treating elderly and poor performance status patients; oral fluoropyrimidines as well as management of resectable metastasis.

## What is an appropriate primary end point in advanced CRC trials?

Improvement in overall survival (OS) has traditionally been regarded as the most important end point in assessing experimental therapy. Yet reliant on this end point may require many years of follow-up and may delay the introduction of effective treatment into routine clinical practice. Furthermore, with effective post-trial treatment, the beneficial effect of experimental therapy may be diluted, especially if the experimental therapy is made available to the trial patients after failing control treatment. Intermediate end points, in particular progression-free survival (PFS), have been generally used as a surrogate for OS. Indeed, in a recent pooled analysis of 39 randomised controlled trials (RCTs) of first line therapy (Tang *et al*, 2007), there was a strong relationship between hazard ratios for PFS and OS. A novel therapy, which produced a 10% reduction in risk of progression would yield an estimated 5.4±1% reduction in risk of death. However, reliance on PFS in assessing a novel treatment effect is not without pitfalls (Panageas *et al*, 2007). The date with radiological progression first evident is often used as a proxy for the true progression, when in fact the true progression time lies somewhere between this date and the last radiological assessment date. As a result, the protocol-specified time interval between radiological assessments used in clinical trials (for example, every 6 weeks *vs* every 12 weeks) may have an impact on the PFS, thus making cross-trial comparisons of clinical benefits with treatment particularly problematic. In addition, definition of PFS is also not universal among phase III trials and this potentially leads to different magnitudes of benefit from the same agent (for example, bevacizumab) seen in advanced CRC (Hurwitz *et al*, 2004; Saltz *et al*, 2008).

## Angiogenesis

Vascular endothelial growth factor (VEGF) represents one of the most important pro-angiogenic proteins. Bevacizumab is a humanised monoclonal antibody against VEGF. A series of randomised studies has initially established and subsequently refined the role of bevacizumab and anti-angiogenic therapy as treatment for advanced CRC. Table 1 shows the efficacy results of these studies (Kabbinavar *et al*, 2003, 2005a, 2005b; Hurwitz *et al*, 2004; Giantonio *et al*, 2007; Hecht *et al*, 2007, 2009; Kohne *et al*, 2007; Saltz *et al*, 2007, 2008; Berry *et al*, 2008; Cunningham *et al*, 2008; Reinacher-Schick *et al*, 2008; Grothey *et al*, 2008b; Tol *et al*, 2009).

Initially a randomised phase II study compared bolus 5-FU/leucovorin (LV) alone with 5-FU/LV combined with two different doses of bevacizumab (5 and 10 mg kg^−1^ every 2 weeks) (Kabbinavar *et al*, 2003). Interestingly, only the lower dose of bevacizumab (5 mg kg^−1^) significantly improved the objective response rate (ORR) and time to tumour progression (TTP) over chemotherapy alone. As a result, this lower dose was chosen in the pivotal study, although there is still much debate about the optimal dose of bevacizumab in solid tumours (Hurwitz *et al*, 2004; Sandler *et al*, 2006; Giantonio *et al*, 2007; Miller *et al*, 2007). The pivotal study showed a significant improvement in ORR, PFS and OS with the addition of bevacizumab to irinotecan/bolus 5-FU/leucovorin (IFL) compared to IFL alone (Hurwitz *et al*, 2004), although it is now recognised that IFL was not an optimal chemotherapy platform in advanced CRC (Fuchs *et al*, 2007). Bevacizumab plus 5-FU/LV also showed a non-significant trend towards better survival compared with IFL alone (Hurwitz *et al*, 2005). Notably this pivotal bevacizumab study only included patients with performance status (PS) 0 or 1. Another randomised trial was performed in patients deemed to be unsuitable for first line irinotecan-based combination chemotherapy regimens (Kabbinavar *et al*, 2005b). In addition, they were required to have at least one of the following characteristics: age ⩾65 years, PS 1 or 2, serum albumin ⩽3.5 g dl^−1^ or prior radiotherapy to abdomen or pelvis. In this study, patients were randomised to receive either 5-FU/LV/bevacizumab or 5-FU/LV/placebo. The addition to bevacizumab to 5-FU/LV resulted in a non-significant prolongation of survival. To more reliably quantify the benefit of adding bevacizumab to 5-FU/LV, the above studies were pooled (Kabbinavar *et al*, 2005a). There was an improvement for 5-FU/LV/bevacizumab over control group (5-FU/LV or IFL) in terms of OS, PFS and ORR.

Most recently, a large RCT (NO16966) was published (Saltz *et al*, 2008). Although the addition of bevacizumab to oxaliplatin–fluoropyrimidine chemotherapy significantly improved PFS compared with oxaliplatin–fluoropymidines alone, no significant differences were seen in terms of ORR and OS. The magnitude of benefit was less than expected from previous studies. One of the reasons cited for the relative small survival benefit for bevacizumab in the NO16966 study was the fact that large proportion of patients (71%) discontinued treatment due to non-progression events (Saltz *et al*, 2008) with many patients stopping oxaliplatin/fluoropyrimidines and bevacizumab due to adverse events. Similar proportion (71%) of patients from the FOLFOX + bevacizumab control arm in PACCE study also stopped treatment due to non-progression events (Hecht *et al*, 2009), whereas 64% of patients did so in the German AIO study (Reinacher-Schick *et al*, 2008). With preclinical data suggesting rapid tumour blood vessel regrowth following cessation of VEGF inhibition (Mancuso *et al*, 2006), one may advocate the continuation of bevacizumab alone until disease progression in the event of cytotoxic drug-induced adverse events. However, re-introduction of VEGF inhibition resulted in the same degree of reduced tumour vasculature as initial VEGF inhibition, suggesting much of the regrown tumour vasculature was still VEGF-dependent (Mancuso *et al*, 2006). Similar observations were also made clinically (Cacheux *et al*, 2008). There is currently no definitive direct clinical evidence to support the necessity of continuing bevacizumab when chemotherapy needs to be stopped due to adverse events. Some preliminary published data support continuing bevacizumab beyond disease progression when second and subsequent lines of chemotherapy were instituted, suggesting a role of continued suppression of the VEGF pathway (Grothey *et al*, 2008b). However, the improved survival seen with continuing bevacizumab beyond disease progression seen in this observational study might only reflect a fitter group of patients being retreated with combination chemotherapy, rather than bevacizumab-specific (Kopetz and Abbruzzese, 2009). Therefore, these non-randomised data should be viewed as hypothesis generating and need confirmation in a randomised trial setting. Currently South West Oncology Group 0600 Trial is testing this hypothesis and until results from this RCT are available, first line use of bevacizumab should be discontinued at the time of disease progression.

Another large study evaluated bevacizumab in a second line setting (Giantonio *et al*, 2007). In patients previously treated with irinotecan and fluoropyrimidine, the addition of bevacizumab to oxaliplatin-infused 5-FU/leucovorin (FOLFOX) significantly improved ORR, PFS and OS compared with FOLFOX alone. However, bevacizumab monotherapy was ineffective in this situation and should not be used routinely.

Tyrosine kinase inhibitors (TK1s) targeting at least partly VEGF have recently been shown to be effective in other solid tumours (Demetri *et al*, 2006; Escudier *et al*, 2007; Motzer *et al*, 2007). Several oral anti-angiogenesis inhibitors have also entered clinical development in CRC. Among these, vatalanib underwent phase III trial testing in both first and second line treatment. In both of these studies, no improvement in efficacy was seen with adding vatalanib to FOLFOX chemotherapy (Hecht *et al*, 2007; Kohne *et al*, 2007).

## Epidermal growth factor receptor

The EGFR-signalling pathway regulates the processes involved in cell differentiation, proliferation, migration, angiogenesis and apoptosis, all of which become dysregulated in cancer cells. Cetuximab is a chimeric monoclonal antibody that specifically targets EGFR with high affinity. After the initial pivotal randomised phase II BOND study which demonstrated the ability of cetuximab to circumvent chemotherapy resistance (Cunningham *et al*, 2004), a series of randomised phase II–III trials for EGFR-targeted monoclonal antibodies (mAbs) have been reported. Table 2 shows the results of these trials (Cunningham *et al*, 2004; Jonker *et al*, 2007; Tejpar *et al*, 2007; Van Cutsem *et al*, 2007, 2009; Borner *et al*, 2008; Ciuleanu *et al*, 2008; Heinemann *et al*, 2008; Sobrero *et al*, 2008; Wilke *et al*, 2008; Bokemeyer *et al*, 2009; Hecht *et al*, 2009). All these studies supported the biological activity of cetuximab in advanced CRC. The benefit of adding cetuximab to first line FOLFIRI in prolonging PFS was relatively small and no improvement in OS results was seen (Van Cutsem *et al*, 2009). In the second line setting, cetuximab/irintoecan significantly improved ORR and PFS (Sobrero *et al*, 2008), but with the commercial availability of cetuximab to patients in the irinotecan control arm on disease progression during the trial, no benefits were seen with OS, although other factors might have contributed to the lack of OS improvement. Forty-seven percent of patients in the control arm received subsequent cetuximab and had a median survival of 13 months, identical to patients who were randomised to irinotecan plus cetuximab and received subsequent treatment without cetuximab (Sobrero *et al*, 2008). One must therefore balance the adverse, but manageable effect of prolonged skin rash with some improvement in remaining progression-free and improvement in at least some domains of quality of life (QoL). In a chemotherapy–refractory situation, cetuximab did show statistically significant improved survival and QoL over best supportive care (BSC) (Jonker *et al*, 2007), but the cost-effectiveness of this approach will need to be carefully evaluated. Notably, no crossover was allowed in the BSC arm to receive cetuximab on disease progression.

Panitumumab, a fully human monoclonal antibody against EGFR was also evaluated against BSC (Van Cutsem *et al*, 2007). Although a significant improvement in PFS was seen with panitumumab, a large proportion of patients (76%) in the BSC arm crossed over to the panitumumab arm on disease progression and precluded any OS benefit to be seen. Nevertheless, this improvement in PFS led to the licensing of panitumumab by the Food and Drug Administration in September 2006. In Europe, the same data was originally rejected for licensing of panitumumab within the European Union. However, with further data available for *K-ras* (Kirsten rat sarcoma viral oncogene homologue) mutation in this study (Amado *et al*, 2008), the licensed indication for panitumumab within EU is treatment of patients with metastatic colorectal carcinoma after failure of fluoropyrimidine-, oxaliplatin-, and irinotecan-containing chemotherapy regimens whose tumours contain non-mutated (wild-type) *K-ras*.

EGFR TKI currently has no role in advanced CRC with only two randomised studies showing little clinical benefit (Rothenberg *et al*, 2005; Santoro *et al*, 2008). Several phase II studies found little additional benefit of EGFR TKI on a conventional chemotherapy platform (Hofheinz *et al*, 2006; Chau *et al*, 2007; Gelibter *et al*, 2007; Zampino *et al*, 2007; Cascinu *et al*, 2008; Fisher *et al*, 2008; Stebbing *et al*, 2008). More importantly, excessive toxicities were encountered in a number of these studies, especially with irinotecan combinations. The lack of EGFR mutations in CRC and supra-additive toxicity of EGFR TKI to chemotherapy regimens may partly explain why development of EGFR TKI in advanced CRC would be unlikely to be fruitful.

With encouraging results seen with individually targeting VEGF and EGFR as successful treatment strategies in advanced CRC, it would be logical to consider dual inhibition of angiogenesis and EGFR with support from preclinical data (Ciardiello *et al*, 2004; Tonra *et al*, 2006). The BOND-2 study showed encouraging results with this approach (Saltz *et al*, 2007). Recruiting similar irinotecan–refractory population to the original BOND study, the BOND-2 study randomised patients between cetuximab plus bevacizumab *vs* irinotecan, cetuximab plus bevacizumab. The efficacy seen with dual inhibition of VEGF and EGFR in the BOND-2 study had improved by 2- to 3-fold in ORR, PFS and OS compared with BOND study, although BOND study had a much larger sample size and this was a cross trial comparison.

However, two large phase III studies have been published disputing the benefit of dual EGFR/VEGF inhibition in combination with chemotherapy (Hecht *et al*, 2009; Tol *et al*, 2009). In the PACCE study, the addition of panitumumab to oxaliplatin-based chemotherapy plus bevacizumab resulted in significantly inferior PFS and OS compared with chemotherapy plus bevacizumab (Hecht *et al*, 2009). A further study, CAIRO 2, also reported a significantly worse PFS with the addition of cetuximab to bevacizumab plus oxaliplatin/capecitabine (CAPOX). No ORR or OS benefit was seen with adding cetuximab in this study (Tol *et al*, 2009). The reasons behind this detrimental effect of adding EGFR antibody to bevacizumab are currently unclear. Additional toxicities were observed with adding panitumumab to bevacizumab/oxaliplatin-based chemotherapy resulting in a lower dose intensity in the PACCE study (Hecht *et al*, 2009). Pharmacokinetic as well as pharmacodynamic interactions could occur between bevacizumab and cetuximab/panitumumab. On the other hand, bevacizumab-associated hypertension, a putative marker for bevacizumab efficacy, was less frequent with CAPOX plus bevacizumab/cetuximab in the CAIRO 2 study (Tol *et al*, 2009). Both PACCE and CAIRO 2 did not pre-select patients with wild-type *K-ras* tumours, the US Intergroup study, CALGB 80405, had amended the entry criteria to exclude patients with *K-ras* mutations and hopefully this would be able to answer definitely whether synergy exists between cetuximab and bevacizumab in wild-type *K-ras* patients.

Aside from combined inhibition of VEGF and EGFR, there are other potential strategies to improve on the efficacy of EGFR-targeted therapy. In a study with patients receiving cetuximab for advanced CRC, 23% of patients were found to have HER2 fluorescent *in-situ* hybridisation positive disease (Finocchiaro *et al*, 2007). Patients with HER2-positive disease had a significantly worse TTP and OS compared to those with HER2-negative disease. Dual targeting treatment is now available for EGFR and HER2 (Geyer *et al*, 2006) and this might be a strategy worth pursuing in advanced CRC.

Preclinical evidence suggested that mAb and TKI against EGFR might not have a completely overlapping mechanism of action and synergistic actions had been observed for administering cetuximab and gefitinib simultaneously in human xenograft models (Matar *et al*, 2004). A phase I study has established that cetuximab and gefitinib can be administered in combination at full individual agent dose in patients who had failed chemotherapy treatment (Baselga *et al*, 2006). Preliminary results showed an encouraging 50% response rate in CRC patients.

## Toxicities from targeted agents

Table 3 and 4 show toxicities seen with agents targeting VEGF and EGFR respectively. Whereas bevacizumab in general does not increase the toxicities from the cytotoxic agents, it does have unique serious side effects, which thankfully are uncommon. However, awareness about hypertension, thromboembolism, bowel perforation and rarely reversible posterior leukoencephalopathy syndrome should be raised to the patients’ primary care physician and other allied health professionals for prompt treatment of these complications. Cetuximab and panitumumab do, however, increase incidences of some side effects (e.g., diarrhoea) from cytotoxic drugs. Nevertheless, integument-related toxicities are very common and may adversely affect patients’ QoL if used on a long-term basis, although oral minocycline may be helpful in some patients (Scope *et al*, 2007). Furthermore, pre-emptive skin treatment (using skin moisturisers, sunscreen, topical steroid and oral doxycycline) starting before panitumumab-based treatment has recently been shown to reduce skin toxicity by >50% with improved QoL compared with reactive skin treatment, that is, starting treatment after development of skin rash (Lacouture *et al*, 2009). There is also a hint that *K-ras* wild-type patients might experience more side effects from cetuximab compared to those treated without cetuximab. The increased toxicity from combining panitumumab and bevacizumab is noteworthy (Hecht *et al*, 2009). However, the CAIRO 2 study did not report any safety concern (Tol *et al*, 2009), further safety data are awaited from cetuximab plus bevacizumab.

## Biomarkers for efficacy and toxicity

Until recently, the most consistent predictor for response and survival to EGFR mAb is the development of skin rash. Multiple RCTs showed a correlation between survival and severity of skin reaction (Cunningham *et al*, 2004; Jonker *et al*, 2007; Van Cutsem *et al*, 2007, 2009). Because no dose-limiting toxicity was observed in phase I studies of cetuximab with the current recommended dosing regimen, individualised dose titration based on the occurrence and severity of skin rash may improve the effectiveness of cetuximab treatment. EVEREST study randomized patients with <grade 2 skin reaction after 3 weeks of cetuximab to either continue on the same dose of cetuximab or escalate dose up to 500 mg m^−2^ (Tejpar *et al*, 2007). Although this study was small, there was nearly a doubling of ORR (16% standard dose *vs* 30% escalating dose). However, due to the small sample size, 95% confidence interval for the ORR overlapped between the two arms. Furthermore, PFS and OS did not show any improvement in dose escalation of cetuximab.

However, skin rash could only be assessed after treatment had been commenced. More than 90% of patients destined to develop rash would only do so after 4 weeks of cetuximab (i.e. after four infusions already) (Jonker *et al*, 2007). Other biomarkers that could predict efficacy before commencing on cetuximab or panitumumab would be more desirable. A number of RCTs evaluating panitumumab/cetuximab has reported their data on a *K-ras* analysable population. Table 5 shows the results of these studies (Amado *et al*, 2008; Karapetis *et al*, 2008; Tejpar *et al*, 2008; Van Cutsem *et al*, 2009; Bokemeyer *et al*, 2009; Hecht *et al*, 2009; Tol *et al*, 2009). *K-ras* mutation occurred in about 35–43% of patients. Patients with wild-type *K-ras* and treated with panitumumab or cetuximab enjoyed generally longer PFS and better ORR compared with those not treated by these antibodies, but those patients with mutant *K-ras* did not derive any benefit from panitumumab/cetuximab. As all of these studies reported *K-ras* data as a retrospective subgroup analysis, no OS benefit has been demonstrated yet in *K-ras* wild-type patients receiving chemotherapy plus cetuximab/panitumumab over those receiving chemotherapy alone. This might be due to underpowered sample sizes in these subgroup analyses. With these emerging data, patients should be tested for *K-ras* mutation before commencing on cetuximab/panitumumab treatment and only those with wild-type tumours should be started on such treatment. Facilities to test for *K-ras* mutation in routine clinical practise are lacking in many institutions. Quality assurance for such testing would be required and central reference laboratories with rapid turnover would be essential, similar to HER 2 testing (Perez *et al*, 2006).

*K-ras* mutation appeared to have no impact on patients treated with bevacizumab. The ORR, PFS and OS benefits of adding bevacizumab to chemotherapy were independent to *K-ras* mutation status (Hurwitz *et al*, 2009). Interestingly, despite patients with *K-ras* wild-type tumours could benefit from cetuximab/panitumumab, when these patients were treated with oxaliplatin-based chemotherapy plus bevacizumab plus cetuximab/panitumumab, no additional benefit was seen over chemotherapy plus bevacizumab (Hecht *et al*, 2009; Tol *et al*, 2009). Indeed they appeared to have worse OS outcome with panitumumab (Hecht *et al*, 2009). For patients with *K-ras* mutant tumours, treatment with CAPOX plus bevacizumab plus cetuximab resulted in worse survival outcome (Tol *et al*, 2009), similar to other studies where chemotherapy plus cetuximab had the worst outcome in *K-ras* mutant patients (Van Cutsem *et al*, 2008; Bokemeyer *et al*, 2009). Therefore, for *K-ras* mutant patients, it would appear to be potentially harmful to treat them with EGFR-targeted therapy.

Further biomarkers have also been evaluated to predict responsiveness to cetuximab/panitumumab. *BRAF* mutation had been found to be mutually exclusive to *K-ras* mutation and *BRAF* mutation was found in 11–14% of *K-ras* wild type patients (Di Nicolantonio *et al*, 2008; Cappuzzo *et al*, 2008b). Patients with *K-ras* wild-type tumours but harbouring *BRAF* mutations did not show any responses to cetuximab/panitumumab and had inferior survival compared to those without *BRAF* mutations (Di Nicolantonio *et al*, 2008; Cappuzzo *et al*, 2008b). In another retrospective study, nuclear factor kappa B positivity by immunohistochemistry also appeared to have worse ORR, PFS and OS in irinotecan-refractory patients receiving irinotecan plus cetuximab (Scartozzi *et al*, 2007), whereas patients with EGFR gene amplification were more likely to respond to cetuximab/panitumumab (Moroni *et al*, 2005; Lievre *et al*, 2006; Sartore-Bianchi *et al*, 2007; Personeni *et al*, 2008; Cappuzzo *et al*, 2008a).

For conventional cytotoxics, a large number of studies has been performed evaluating variations in genes associated with drug metabolism and targets and the effects of these variations on treatment outcome and toxicities. This has been systematically reviewed (Funke *et al*, 2008). Most of these studies were small (<200 patients), retrospective and non-randomised; included a heterogeneous patient population and utilised a variety of laboratory techniques and biological materials including primary tumours, metastasis and peripheral blood. Few genetic variants have therefore been shown to be unequivocally associated with treatment outcome. Overall, the homozygous UGT1A1^*^28 insertion polymorphism was associated with increased risk of irinotecan-related toxicities. XPD gene (ERCC 2) variations led to differences in DNA-repair capability. Glutathione-*S*-transferases (GST) are phase II metabolising enzymes involved in detoxification of platinum compounds. GSTP1-105 mutations were associated with improved outcome (Funke *et al*, 2008).

Recently, the largest published RCT in advanced CRC, FOCUS (Seymour *et al*, 2007a), reported the first results of a nested prospective search for biomarkers within the FOCUS study (Braun *et al*, 2008). Topo 1, a molecular target of SN38 (active metabolite of irinotecan) was found to be a predictive biomarker to irinotecan therapy in the assessable 1313 patients. Patients with low Topo 1 did relatively well with first line 5-FU monotherapy, but did not benefit in PFS or OS from adding irinotecan or oxaliplatin. With increasing expression of Topo 1, the outcome with 5-FU alone was worse, but addition of a second drug improved the treatment outcome, with a major improvement in survival for the highest expressing patients. This observation was seen with the addition of either irinotecan or oxaliplatin, but the association with improved survival was stronger with irinotecan. None of the other biomarkers studied, including ERCC1, MLH1/MSH2, p53, MGMT, COX-2 protein expression as assessed by tumour immunohistochemistry or GST-P1, ABCB1, XRCC1, ERCC2, UGT1A1 germ-line polymorphism as assessed by macrodissected normal tissue, were found to be associated with treatment outcome from 5-FU plus either irinotecan or oxaliplatin (Braun *et al*, 2008). Within the same group of patients in FOCUS, those with KRAS and/or BRAF mutation had a significantly worse OS compared to patients with no mutation. However, treatment efficacy from oxaliplatin or irinotecan was not impacted by the KRAS/BRAF mutation status (Richman *et al*, 2008).

## Should oral fluoropyrimidines substitute infused fluorouracil in advanced CRC?

Only capecitabine has been evaluated as combination treatment regimens in randomised phase III trials in conjunction with oxaliplatin, irinotecan±bevacizumab. Such data are currently lacking with UFT and S-1. Five phase III RCTs have been reported to establish non-inferiority of CAPOX compared with FOLFOX. Table 6 shows the efficacy results of these studies (Diaz-Rubio *et al*, 2007; Ducreux *et al*, 2007; Porschen *et al*, 2007; Cassidy *et al*, 2008; Rothenberg *et al*, 2008). Two studies did not meet the primary objective of demonstrating non-inferiority in PFS with CAPOX compared with FOLFOX (Diaz-Rubio *et al*, 2007; Porschen *et al*, 2007). In the third study (Ducreux *et al*, 2007), a rather permissive non-inferiority margin was used with a primary end point being ORR – a questionable primary efficacy end point for first line advanced CRC trials in the modern era. However, the largest study, NO16966 (a commercially sponsored study), did clearly establish non-inferiority in PFS with CAPOX compared with FOLFOX, although the convenience of capecitabine did come with a price of nearly doubling of grade 3/4 diarrhoea (20% CAPOX *vs* 11% FOLFOX) in the dose schedule used in NO16966 (Cassidy *et al*, 2008). A meta-analysis of the above studies plus two further randomised phase II studies reported a significantly reduced ORR with CAPOX compared with FOLFOX (Arkenau *et al*, 2008). However, CAPOX was non-inferior in PFS and OS compared with FOLFOX.

Two studies have also been reported comparing capecitabine/irinotecan (CAPIRI) with FOLFIRI – both of which did not reach their recruitment targets. In the first EORTC 40015 study, recruitment was suspended after 85 patients (originally planned recruitment *n*=629) because of the frequent occurrence of grade 3/4 diarrhoea (CAPIRI 37% *vs* FOLFIRI 13%) and more fatal events occurring in the CAPIRI arm (CAPIRI *n*=6 *vs* FOLFIRI *n*=2). Five deaths in the CAPIRI arm and both deaths in the FOLFIRI arm were considered to be treatment-related. PFS and OS were all worse with CAPIRI compared with FOLFIRI (Kohne *et al*, 2008). In the second study BICC-C (Fuchs *et al*, 2007), CAPIRI was associated with a significantly worse PFS compared with FOLFIRI, when associated with higher rates of severe vomiting, diarrhoea and dehydration. In view of the toxicity concerns, further enrolment into CAPIRI arm in this study was discontinued after the first period of the study (pre-bevacizumab) with 430 patients randomised. However, both the EORTC 40015 and BICC-C had one further complicating factor – a second randomisation to either celecoxib or placebo. Coxibs have been associated with an increased risk of cardiovascular thrombotic events in colorectal neoplasia (Solomon *et al*, 2005; Kerr *et al*, 2007). There might be an interaction between celecoxib with CAPIRI that compromised CAPIRI's efficacy and increased its toxicity. A further large randomised study (CAIRO) evaluating CAPIRI completed patient recruitment (Koopman *et al*, 2007). CAPIRI treatment did result in grade 3–4 diarrhoea incidence of 27%. A further randomised study of CAPOX plus bevacizumab *vs* CAPIRI plus bevacizumab using a lower dose of capecitabine and irinotecan resulted in a more tolerable grade 3–4 rate of 16 (CAPOX) and 13% (CAPIRI) respectively (Reinacher-Schick *et al*, 2008).

Taken together, when using an irinotecan-based regimen in the treatment of first line metastatic CRC, FOLFIRI is the preferred approach unless there is a clear contraindication to continuous infusion 5-FU. Further development in alterative dosing schedule of CAPIRI could provide a better efficacy and safety profile than that used in these three published trials. When using an oxaliplatin-based regimen, capecitabine could substitute infused 5-FU. However, the relative benefit/cost-effectiveness may also depend on the health care system and reimbursement pattern of individual countries (Mayer, 2007).

## Should we use sequential treatment or first line combination chemotherapy?

In a pooled analysis of 11 phase III trials in CRC including 5768 patients (Grothey and Sargent, 2005), there was a strong correlation between improved OS and percentage of patients treated with 5-FU/LV, irinotecan and oxaliplatin at some point in their disease. However, combination doublet therapy was not always beneficial in the first line treatment of advanced CRC. Although this analysis was not a formal meta-analysis using individual patient data, it gave a timely indication to clinicians of the importance of having access to all three active drugs – fluoropyrimidines, irinotecan and oxaliplatin in advanced CRC. Several RCTs had attempted to determine whether upfront combination chemotherapy offers any advantage over giving these agents in a sequential manner. Table 7 shows the results of three studies (Koopman *et al*, 2007; Seymour *et al*, 2007a; Cunningham *et al*, 2009).

FOCUS trial is the largest RCT conducted to date in advanced CRC (Seymour *et al*, 2007a). 2135 patients were randomly allocated into strategy (A) sequential single agent 5-FU/LV followed by single agent irinotecan; strategy (B) single agent 5-FU/LV followed by combinations with either FOLFIRI or FOLFOX and strategy (C) first line combination treatment with FOLFIRI or FOLFOX and then the reverse regimen on disease progression. Strategies B and C produced very similar survival, both slightly better than strategy A, but no significant OS differences were seen among all three strategies (*P*>0.01). Similar to other RCTs (Tournigand *et al*, 2004), comparisons of irinotecan *vs* oxaliplatin, whether used in first line, second line combinations or at any time showed no significant OS difference in FOCUS trial. However, median survival in the FOCUS trial appeared to be lower than other contemporary studies, possibly due to the fact that only patients with unresectable metastasis were recruited and only 23% of patients had received all three active drugs of fluorouracil, irinotecan and oxaliplatin. Again similar to other trials (Hospers *et al*, 2006; Cunningham *et al*, 2009), ORR and PFS for first line FOLFOX and FOLFIRI were significantly better than for fluorouracil alone, but this was achieved with the expense of greater toxicity. There appeared to be no advantage or disadvantage in QoL associated with first line combination treatment.

Another study (CAIRO) randomly allocated patients to sequential capecitabine followed by irinotecan followed by CAPOX (sequential arm) or first line CAPIRI followed by second line CAPOX (combination arm) (Koopman *et al*, 2007). Again combination treatment did not significantly improve OS over sequential treatment, despite an improvement in ORR and PFS with first line combination treatment. Interestingly, the deterioration in QoL functioning was on average more for combination treatment in all domains in this study. LIFE study randomly allocated patients to sequential LV5FU2 followed by irinotecan or FOLFOX followed by irinotecan. Upfront combination FOLFOX significantly improved response rate and PFS, but no improvement of OS was seen over sequential treatment (Cunningham *et al*, 2009).

A fourth study addressed the same issue in the elderly or physically unfit population (FOCUS 2) (Seymour *et al*, 2007b). The study used a 2 × 2 factorial design to assess firstly whether capecitabine gave better QoL improvement compared with 5-FU, reserving oxaliplatin combination for second line treatment. Second comparison assessed whether addition of oxaliplatin to either capecitabine or 5-FU in first line setting would improve PFS over single agent. This study also commenced with a reduced starting dose of 80% standard dose. With a median age of 75 and 30% of patients with PS 2, this represented an older and frailer population compared with other RCTs. Only 30–50% of patients escalated to a 100% dose. Addition of oxaliplatin increased ORR (*P*<0.0001), but did not significantly improve PFS (*P*=0.06) or OS (*P*=0.61). In this patient population, substituting 5-FU with capecitabine did not result in any significant differences in PFS or OS. Interestingly in some measures of QoL, capecitabine-containing regimen was worse than infused 5-FU. Capecitabine also led to significantly increased incidences of nausea, diarrhoea, lethargy and hand foot syndrome.

Currently in patients with unresectable metastasis, it would be reasonable to consider first line monotherapy to maintain QoL, but these patients must be monitored closely during treatment in order not to miss the therapeutic window for exposure to other active agents. However, both FOCUS and CAIRO studies utilised treatment strategies without bevacizumab and cetuximab and thus support for sequential treatment might not apply for patients with access to these biological agents. On the other hand, there have been no RCT to demonstrate OS benefit to give combination chemotherapy plus monoclonal antibody over monotherapy plus monoclonal antibody in a sequential manner. For patients with resectable metastasis and perhaps those with heavy tumour burden or significant symptoms, they might benefit more with combination first line chemotherapy to achieve higher and more durable treatment responses.

## What is the optimal duration of treatment?

Although the optimal duration of adjuvant chemotherapy has been addressed in CRC (O’Connell *et al*, 1998; Chau *et al*, 2005; Andre *et al*, 2007), randomised data are lacking in advanced CRC comparing the two strategies of continuous treatment until disease progression or defined treatment duration. The United Kingdom Medical Research Council published a randomised study comparing intermittent or continuous palliative first line chemotherapy for 354 patients with advanced CRC (Maughan *et al*, 2003). No survival differences were found between the two treatment strategies, though intermittent therapy was associated with reduced toxicity. Notably, despite being a principal intention of the trial, only 66 (37%) patients randomly assigned to the intermittent group was rechallenged with the same first line chemotherapy.

With the advent of widespread first line use of oxaliplatin-based chemotherapy, oxaliplatin-induced cumulative neuropathy is becoming a significant clinical problem. It can cause substantial impairment of patients’ QoL as well as potentially compromising efficacy due to reduced dose intensity. Randomised trials have so far suggested potential benefits of calcium/magnesium infusion, glutamine and glutathione in preventing oxaliplatin-induced peripheral neuropathy (Wolf *et al*, 2008), but few drugs are effective to treat established peripheral neuropathy. One of the strategies that had been tested in a phase III setting to address this issue was the ‘stop and go’ strategy. The OPTIMOX 1 study randomised 620 patients to FOLFOX 4 till disease progression or FOLFOX 7 (high dose of oxaliplatin and omission of bolus 5-FU) for 12 weeks followed by LV5FU2 followed by oxaliplatin reintroduction at the time of disease progression (Tournigand *et al*, 2006). Overall, no differences were seen in response rates, durations of disease control or overall survival between the two arms, but the incidence of neurotoxicity was markedly reduced in the FOLFOX 7 stop and go arm during oxaliplatin omission phase of LV5FU2, suggesting a novel way to reduce toxicity for patients. However, large variations among treatment centres in reintroducing oxaliplatin might have explained the lack of efficacy differences between the two arms as oxaliplatin reintroduction had a significant positive impact on survival (de Gramont *et al*, 2007).

A further study from the United States followed similar trial design of assessing intermittent oxaliplatin *vs* continuous oxaliplatin in the FOLFOX plus bevacizumab regimen (Grothey *et al*, 2008a). This study also assessed the use of calcium/magnesium infusion in a 2 × 2 factorial design. However, this study was discontinued early due to an unplanned interim analysis of ORR showing worse results with patients receiving calcium/magnesium infusion based on data collected through the clinical research organisation. These inferior results with calcium/magnesium infusion were not confirmed subsequently by either investigator-reported or centrally reviewed ORR. Interestingly, in this study, intermittent oxaliplatin was associated with a significant prolongation of time to treatment failure as well as PFS.

Following on from the OPTIMOX study, a randomised phase II study was performed evaluating the OPTIMOX 1 strategy *vs* FOLFOX 7 for 3 months only and then reintroduced FOLFOX 7 on disease progression (thus a complete chemotherapy-free period) (Maindrault-Goebel *et al*, 2007), there was no significant differences in OS, PFS, ORR or duration of disease control between the two arms, although there was a trend towards a benefit with continuous chemotherapy. However, this may simply be a reflection that 3 months of initial chemotherapy were not sufficient and patients should be treated for longer periods (at least 6 months) before contemplating a treatment break. Another GISCAD study randomised 266 patients to either intermittent FOLFIRI (alternating FOLFIRI for 2 months and stopping chemotherapy for 2 months) or continuous FOLFIRI till disease progression (Mandala *et al*, 2009). Once again, there were no significant differences in ORR, PFS or OS between the two strategies. Interestingly, patients treated with intermittent FOLFIRI had a reduced risk of venous thromboembolism – a complication with significant impact on patients’ QoL (Mandala *et al*, 2009). A large phase III COIN trial addressing this issue has finished recruiting 2421 patients into a three arm comparison with one of the arms being intermittent treatment schedule *vs* control continuous treatment schedule.

For second line treatment, one study randomised patients to stop after 6 months of irinotecan or continuous irinotecan until disease progression (Lal *et al*, 2004). Again no survival differences were seen between these two strategies, although only a small proportion (17%) of patients was progression-free after 6 months of irinotecan, thus eligible for randomisation. Nevertheless, there was no detriment to QoL for those patients who continued irinotecan after an appropriate dose reduction in the initial phase of treatment.

There is currently no detrimental survival effect for treatment for a defined duration (at least 6 months) followed by a treatment break compared with continuous treatment until disease progression. Prolonged continuous treatment may be associated with side effects such as venous thromboembolism.

## How do we treat the elderly, poor performance status or asymptomatic patients?

Elderly patients represent an increasing challenge. Declining organ reserve may lead to an increased risk and decreased tolerance to chemotherapy-induced side effects. However, recent pooled analyses on elderly patients (aged 70 or more) with both oxaliplatin-based and irinotecan-based chemotherapy showed similar benefit from chemotherapy in terms of ORR, PFS and OS compared with those aged <70 years (Goldberg *et al*, 2006; Folprecht *et al*, 2008). Toxicity was similar when treated with irinotecan-based chemotherapy, but more neutropenia, thrombocytopenia was seen in the elderly when treated with FOLFOX. Caution needs to be exercised to extrapolate these data to routine clinical practise, as patients enrolled into these RCTs were fit older patients and only 0.9–2% of patients were octogenarians. Similar efficacy and toxicity were observed when older patients were treated with bevacizumab-based treatment compared with younger patients (Kabbinavar *et al*, 2009).

There is often uncertainty of whether patients with poor PS would benefit from the treatment to the same extent to patients with better PS. In another pooled analysis of nine first line chemotherapy RCTs (Sargent *et al*, 2009), patients with PS 2 had a significantly worse PFS and OS compared to those with PS 0 or 1. However, the likelihood of benefiting from experimental treatment was similar between different PS groups. Furthermore, patients with PS 2 also benefited with similar magnitude from combination therapy over monotherapy compared to patients with PS 0 or 1. Patients with PS 2 did experience more nausea or vomiting, but otherwise had no increase in other adverse events. Any differential toxicity in experimental *vs* control treatment was not PS-dependent. Once again, these data cannot be extrapolated to patients with PS 3 or 4 who were excluded from such RCTs and therefore should not be offered chemotherapy routinely.

In conjunction with the previously mentioned FOCUS 2 study, which recruited elderly or poor PS patients, sequential or combination strategies are both reasonable in these patients and there is no evidence that efficacy is compromised or toxicity more pronounced in these groups of elderly or PS 2 patients.

For patients with unresectable but low volume, asymptomatic disease, there is some controversy about whether treatment needs to be initiated immediately or whether an expectant policy can be adopted for a period of time. Whereas the original Nordic study concluded that early treatment in asymptomatic patients with advanced CRC prolonged survival, asymptomatic period and time to progression (1992), a meta-analysis of two subsequent studies conducted in Canada and Australasia did not show significant improvement in survival and QoL to commence early treatment in asymptomatic patients (Ackland *et al*, 2005). Notably, the latter two studies terminated prematurely due to poor accrual. It is unlikely further studies would be performed to address this issue. However, biological agents as monotherapy with relatively fewer side effects to conventional cytotoxic treatment might be considered to stabilise the disease (Pessino *et al*, 2008) and delay the introduction of combination cytotoxic drugs with biological agents.

## What are the current controversies with resection of colorectal metastasis?

Aggressive surgical approaches to metastatic disease are increasingly practised with a proportion of patients enjoying long-term survival. Five-year survival rates of 30–40% are seen with resection of liver metastasis (Fernandez *et al*, 2004), despite a lack of randomised data to support surgery. Introduction of new drugs such as oxaliplatin and more recently monoclonal antibodies have allowed sufficient downsizing of ‘unresectable’ liver metastases to convert them to resectable following therapy.

In patients with resectable liver metastasis, the role of peri-operative chemotherapy is still controversial. The European Organisation for Research and Treatment of Cancer (EORTC) 40983 study randomised 364 patients to either peri-operative FOLFOX or surgery alone (Nordlinger *et al*, 2008). Ninety-two percent of patients had 1–3 liver metastasis and 75% had >2 years between original diagnosis and development of liver metastasis. Three-year PFS benefit from peri-operative FOLFOX did not reach the conventional level of significance in all randomised patients (*P*=0.058; absolute difference: 7.2%), although 3-year progression-free survival was significantly improved in those receiving peri-operative FOLFOX in the eligible population (*P*=0.041; absolute difference in 3-year PFS: 8.1%) and in the resected patients (*P*=0.025; absolute difference in 3-year PFS: 9.2%).

For patients who are considered to have inoperable liver metastasis, a proportion of patients would achieve sufficient downsizing after a period of conversion chemotherapy to allow liver resection. In one study, 13% of patients were converted from unresectable to resectable after chemotherapy (Adam *et al*, 2004). Although OS was significantly worse in this group of patients (*P*=0.01) compared with those who were primarily resectable, this former group of initially unresectable patients still had a respectable 5-year OS rate of 33%.

The rate of liver resection correlated significantly with the ORR of neoadjuvant chemotherapy (Folprecht *et al*, 2005). FOLFOXIRI (5-FU/LV/oxaliplatin/irinotecan) resulted in a higher response rate compared with FOLFIRI (60 *vs* 34% respectively; *P*<0.0001) (Falcone *et al*, 2007). This improved response rate led to an increased rate of surgical resection of metastasis. R0 resection was achieved in a higher proportion of patients receiving FOLFOXIRI, which might have contributed to the significant improvement of PFS and OS with FOLFOXIRI compared with FOLFIRI. The addition of cetuximab to FOLFIRI also significantly improved ORR and thus R0 resection of metastasis compared with FOLFIRI alone (4.8 *vs* 1.7% respectively; *P*=0.002) (Van Cutsem *et al*, 2009). This improvement in ORR with cetuximab was even more pronounced in the *K-ras* wild-type population (Van Cutsem *et al*, 2008). In the subgroup of patients with liver metastasis only, R0 resection was increased and PFS significantly improved when cetuximab was added to FOLFIRI. Although bevacizumab did not significantly improve ORR when added to oxaliplatin/fluoropyrimidine compared with oxaliplatin/fluoropyrimidine alone in the NO16966 study, there was a numerical increase in the curative surgery rate in the bevacizumab-containing arm (19.2 *vs* 12.9%), although this was a *post hoc* analysis. Even in patients resistant to initial chemotherapy, one study showed that subsequent addition of cetuximab to chemotherapy induced a response and allowed 12% of patients to proceed to surgery with a median OS of 20 months (Adam *et al*, 2007) and no increase in peri-operative mortality. Currently there is no universally agreed optimal conversion chemotherapy before resection of liver metastasis. FOLFOXIRI or chemotherapy plus cetuximab in *K-ras* wild-type patients represent attractive options.

Liver injury secondary to chemotherapeutic agents is increasingly recognised. Hepatic vascular lesions could be seen more frequently in patients receiving neoadjuvant oxaliplatin-based chemotherapy (Aloia *et al*, 2006) leading to higher red blood transfusion requirement. In addition, more prolonged neoadjuvant treatment led to a higher rate of re-operation and a longer hospital stay (Aloia *et al*, 2006). Pre-operative irinotecan was associated with steatohepatitis and patients with this liver injury had higher 90-day mortality (Vauthey *et al*, 2006). Neoadjuvant cetuximab was not found to be associated with specific pathological liver damage yet (Adam *et al*, 2007). These studies highlighted the importance of chemotherapy-induced damage on the non-tumour bearing liver – a complication that needs to be carefully assessed in future studies.

Although lung metastasis is less common than liver involvement, similar long-term survival has been observed after complete resection with a 5-year survival rate of 48% in a recent systematic review of 20 surgical retrospective series (Pfannschmidt *et al*, 2007). However, similar to liver resection, it would be difficult to conduct a randomised trial against no resection nowadays. Similar approach of neoadjuvant chemotherapy in liver metastasis may be beneficial in CRC lung metastasis.

## Conclusions

The addition of bevacizumab to chemotherapy improved the efficacy over chemotherapy alone in both first and second line settings, although the magnitude of benefit may not be as great when a more optimal chemotherapy platform is used. Studies performed thus far did not address conclusively whether bevacizumab should be continued in subsequent lines of treatment. Anti-angiogenesis TKI has not shown any additional benefit over chemotherapy alone so far. Although some benefits were seen with cetuximab in all settings of treating advanced CRC, *K-ras* mutation status provides an important determinant of who would not benefit from such treatment. Caution should be exercised when combining anti-angiogenesis with anti-EGFR strategy until further randomised data become available. In totality of randomised evidence, capecitabine is non-inferior to intravenous fluorouracil when combined with oxaliplatin, although not all study results were consistent. On the other hand, the dose schedule used in randomised trials of irinotecan plus capecitabine might be too toxic, hampering the potential use of this combination. In patients with extensive unresectable metastasis, a staged strategy of a single agent followed by a combination treatment might be an alternative to upfront combination treatment, whereas in patients with resectable metastasis, a combination therapy with a high response rate appears to be essential. Sequential or combination strategies are both reasonable in elderly or PS 2 patients. There is no evidence that the efficacy is compromised or toxicity more pronounced in elderly or PS 2 patients. Currently the optimal duration of treatment remains uncertain, but there does not appear to be clearly detrimental effect to stop treatment after a defined duration of at least 6 months.

Management of advanced colorectal cancer has become increasingly complex with our expanding (and improved) array of medical, radiation and surgical treatment. What is certain, however, is that our patients are benefiting from this intense research focusing on colorectal cancer.

## Figures and Tables

**Table 1:** Selected studies evaluating angiogenesis inhibitors in advanced colorectal cancer

**Study**	**Treatment arms**	**Number of patients**	**Response rates (%)**	***P*-value**	**Median progression-free survival (months)**	***P*-value**	**Median overall survival (months)**	***P*-value**
*First line*								
Kabbinavar *et al* (2003)	5-FU/LV	36	17	—	5.2	—	13.8	NR
	5-FU/LV/BEV (5 mgkg^−1^)	35	40	0.029	9.0	0.005	21.5	NR
	5-FU/LV/BEV (10 mgkg^−1^)	33	24	0.434	7.2	0.217	16.1	NR
Hurwitz *et al* (2004)	IFL	411	34.8	—	6.2	—	15.6	—
AVF 2107	IFL/BEV	402	44.8	0.004	10.6	<0.001	20.3	<0.001
	5-FU/LV/BEV	110	40.0	0.66	8.8	0.4192	18.3	0.2521
Kabbinavar *et al* (2005b)	5-FU/LV	105	15.2	—	5.5	—	12.9	—
	5-FU/LV/BEV	104	26.0	0.055	9.2	0.0002	16.6	0.16
Kabbinavar *et al* (2005a)	5-FU/LV or IFL	241	24.5	—	5.55	-	14.6	—
	5-FU/LV/BEV	249	34.1	0.019	8.77	0.0001	17.9	0.0081
Saltz *et al* (2008)	FOLFOX or CAPOX	701	38	—	8.0	0.0023	19.9	0.077
XELOX-1/ NO16966	FOLFOX/CAPOX + BEV	699	38	0.99	9.4		21.3	
								
Tol *et al* (2009)	CAPOX + BEV	368	50	—	10.7	—	20.3	—
CAIRO 2	CAPOX + BEV + cetuximab	368	52.7	0.49	9.4	0.01	19.4	0.16
								
Hecht *et al* (2009)	FOLFOX + BEV	410	48	—	11.4	HR: 1.27	24.5	HR: 1.43
PACCE	FOLFOX + BEV + PAN	413	46	NS	10.0	(95% CI: 1.06–1.52)	19.4	(95% CI: 1.11–1.83)
Hecht *et al* (2009)	FOLFIRI + BEV	115	40	—	11.7	HR: 1.19	20.5	HR: 1.42
PACCE	FOLFIRI + BEV + PAN	115	43	NS	10.1	(95% CI: 0.79–1.79	20.7	(95% CI: 0.77–2.62)
Reinacher-Schick *et al* (2008) a	CAPOX + BEV	127	53	—	10.4	—	26.7	—
AIO 0604	CAPIRI + BEV	120	55	NR	12.1	0.27	Not reached	0.55
Hecht *et al* (2009)	FOLFOX	583	46	—	7.7	—	20.5	—
CONFIRM 1	FOLFOX + PTK/ZK	585	42	NS	9.1	0.108	21.4	0.260
Grothey *et al* (2008b) BriTE^b^	Chemotherapy + BEV (non-randomised US cohort study)	1953	NR	NR	9.9	NR	25.1	NR
Berry *et al* (2008) (BEAT^b^)	Chemotherapy + BEV (non-randomised non-US cohort study)	1914	NR	NR	10.8	NR	22.7	NR
								
*Second line*								
Giantonio *et al* (2007)	FOLFOX	291	8.6	—	4.7	—	10.8	—
ECOG E3200	FOLFOX/BEV (10 mg kg^−1^)	289	22.7	<0.0001	7.3	<0.0001	12.9	0.0011
	BEV (10 mg kg^−1^)	243	3.3		2.7		10.2	
								
Kohne *et al* (2007)	FOLFOX	429	18	—	4.1	—	11.8	—
CONFIRM 2	FOLFOX + PTK/ZK	426	19	NS	5.6	0.026	12.1	0.511
Cunningham *et al* (2008) a	A. FOLFOX+BEV	66	27		7.8	B *vs* A	NR	—
HORIZON I	B. FOLFOX+cediranib (low dose)	71	18	—	5.8	0.29	NR	
	C. FOLFOX+cediranib (high dose)	73	19	NR	7.2	C *vs* A 0.79	NR	NS
Saltz *et al* (2007) a	Irinotecan/cetuximab/ BEV	43	37	—	7.3	—	14.5	—
BOND 2	Cetuximab/ BEV	40	20	NR	4.9	NR	11.4	NR

LV=leucovorin; FOLFOX: oxaliplatin/infused 5-FU/LV; BEV=bevacizumab; CAPOX: capecitabine/oxaliplatin; IFL=irinotecan/bolus 5-FU/LV; FOLFIRI: irinotecan-infused 5-FU/LV; CAPIRI: capecitabine/irinotecan; PAN=panitumumab; NR=not reported; NS=Not significant; HR=hazard ratio; CI=confidence interval.

The first treatment arm of each study was the control arm. Unless stated, all bevacixumab was given at 2.5 mg kg^−1^ per week. All *P*-values were compared with control arms.

a Randomised phase II studies.

b Observational registry studies.

**Table 2:** Randomised studies evaluating epidermal growth factor receptor inhibitors in advanced colorectal cancer

**Study**	**Treatment arms**	**Number of patients**	**Response rates (%)**	***P*-value**	**Median progression-free survival (months)**	***P*-value**	**Median overall survival (months)**	***P*-value**
*First line*								
Van Cutsem *et al* (2009)	FOLFIRI	599	38.7	—	8.0	—	18.6	—
CRYSTAL	FOLFIRI + cetuximab	599	46.9	0.004	8.9	0.048	19.9	0.31
Bokemeyer *et al* (2009) a	FOLFOX	168	36	—	7.2	—	NR	NR
OPUS	FOLFOX + cetuximab	169	46	0.064	7.2	0.62	NR	
Borner *et al* (2008) a	CAPOX	37	14	—	5.8	—	16.5	—
SAKK	CAPOX + cetuximab	37	41	NR	7.2	NR	20.5	NR
Heinemann *et al* (2008) a	CAPIRI +cetuximab	93	47	—	6.7	—	NR	—
German AIO	CAPOX + cetuximab	92	48	NR	7.9	NR	NR	NR
Ciuleanu *et al* (2008) a	FOLFIRI +cetuximab	78	45	—	8.3	—	18.9	—
CECOG	FOLFOX +cetuximab	77	43	NR	8.6	NS	17.4	NR
Hecht *et al* (2009)	FOLFOX + BEV	410	48	—	11.4	HR: 1.27	24.5	HR: 1.43
PACCE	FOLFOX + BEV + PAN	413	46	NS	10.0	(95% CI: 1.06–1.52)	19.4	(95% CI: 1.11–1.83)
Hecht *et al* (2009)	FOLFIRI + BEV	115	40	—	11.7	HR: 1.19	20.5	HR: 1.42
PACCE	FOLFIRI + BEV + PAN	115	43	NS	10.1	(95% CI: 0.79–1.79)	20.7	(95% CI: 0.77–2.62)
								
*Second line*								
Sobrero *et al* (2008)	Irinotecan	650	4.2	<0.0001	2.6	<0.0001	9.99	0.7115
EPIC	Irinotecan + cetuximab	648	16.4		4.0		10.71	
								
*Third and subsequent line*								
Jonker *et al* (2007)	BSC	285	0	<0.001	1.8	<0.001	4.6	0.005
NCIC CO17	Cetuximab + BSC	287	8		1.9		6.1	
Van Cutsem *et al* (2007)	BSC	232	0	<0.0001	1.8	<0.0001	NR	0.81
	Panitumumab + BSC	231	10		2		NR	
Cunningham *et al* (2004)	Cetuximab	111	10.8	0.0074	1.5	<0.001	6.9	0.48
BOND^a^	Irinotecan + cetuximab	218	22.9		4.1		4.8	
Tejpar *et al* (2007) EVEREST^a^	Irinotecan + cetuximab (standard dose)	45	16	—	3.9	—	10	—
	Irinotecan + cetuximab (escalating dose)	44	30	NR	4.8	NR	8.6	NR
Wilke *et al* (2008) MABEL^b^	Irinotecan + cetuximab	1147	20.1	—	3.2	—	9.2	—

LV=leucovorin; FOLFOX=oxaliplatin/infused 5-FU/LV; BEV=bevacizumab; FOLFIRI=irinotecan /infused 5-FU/LV; CAPOX=capecitabine/oxaliplatin; BSC=best supportive care; PAN=panitumumab; NR=not reported; HR=hazard ratio; CI=confidence interval.

The first treatment arm of each study was the control study.

All *P*-values were compared with control arms.

a Randomised phase II studies.

b Observational registry studies.

**Table 3:** Toxicities encountered during selected studies evaluating bevacizumab in advanced colorectal cancer

**Study**	**Treatment arms**	**Number of evaluable patients**	**Grade 3/4 hypertension (%)**	**Venous thrombosis (%)**	**Arterial thrombosis (%)**	**Grade 3/4 bleeding (%)**	**Grade 2-4 proteinuria (%)**	**GI perforation (%)**
Kabbinavar *et al* (2003)	5-FU/LV	35	0	6	3	0	NR	NR
	5-FU/LV/BEV (5 mg kg^−1^)	35	9	26	0	0	NR	NR
	5-FU/LV/BEV (10 mg kg^−1^)	32	25	6	6	9	NR	NR
Hurwitz *et al* (2004)	IFL	397	2	11.4	1	2.5	6.6	0
AVF 2107	IFL/BEV	393	11	12.5	3.3	3.1	3.9	1.5
	5-FU/LV/BEV	109	6.4	9.2	4.6	6.4	1.8^a^	0
Kabbinavar *et al* (2005b)	5-FU/LV	104	3	11	5	3	4	0
	5-FU/LV/BEV	100	16	9	10	5	8	2
Kabbinavar *et al* (2005a)	5-FU/LV or IFL	237	3	9	3	2	4	0
	5-FU/LV/BEV	244	16	10	5	5	9	1
								
								
Giantonio *et al* (2007)	FOLFOX	285	1.8	2.5	0.4	0.4	0	0
ECOG E3200	FOLFOX/BEV (10 mg kg^−1^)	287	6.2	3.4	0.9	3.4	0.7	1
	BEV (10 mg kg^−1^)	234	7.3	0.4	0.4	2.1	0	1.3
Saltz *et al* (2007)	FOLFOX or CAPOX	675	1	5	1	1	NR	<1
XELOX-1/NO16966	FOLFOX or CAPOX/BEV	694	4	8	2	2	<1	<1
Hecht *et al* (2009)	FOLFOX + BEV	397	5	12	NR	NR	NR	0
PACCE	FOLFOX + BEV + panitumumab	407	4	13	NR	NR	NR	0
Hecht *et al* (2009)	FOLFIRI + BEV	113	2	11	NR	NR	NR	NR
PACCE	FOLFIRI + BEV + panitumumab	111	3	24	NR	NR	NR	NR
Tol *et al* (2009)	CAPOX + BEV	366	14.8	6.8	3.3	1.6	NR	0.3
CAIRO 2	CAPOX + BEV + cetuximab	366	9.3	8.2	2.2	0.5	NR	1.6
Berry *et al* (2008) BEAT	Chemotherapy + BEV	1914	5.3	NR	1.5	3.4	1.1	1.8
Grothey *et al* (2007) BriTE	Chemotherapy + BEV	1953	NR	NR	1.8	2.4	NR	1.8

a Only grade 3 toxicity was reported.

LV=leucovorin; FOLFOX=oxaliplatin/infused 5-FU/LV; BEV=bevacizumab; CAPOX=capecitabine/oxaliplatin; IFL=irinotecan/bolus 5-FU/LV; NR=not reported.

**Table 4:** Toxicities encountered during randomised studies evaluating EGFR antibodies in advanced colorectal cancer

**Study**	**Treatment arms**	**Number of evaluable patients**	**Grade 3/4 diarrhoea (%)**	**Grade 3/4 nausea + vomiting (%)**	**Grade 3/4 hypo-magnesiumia (%)**	**Grades 2–4 skin reaction (%)**	**All grades infusion reaction (%)**
CRYSTAL	FOLFIRI	602	10.5	5.0	0.2^a^	0.2	0
	FOLFIRI + cetuximab	600	15.7	4.7	1.8^a^	19.7	2.5
OPUS	FOLFOX	168	7	NR	0	0.6	2
	FOLFOX + cetuximab	170	8	NR	2	18	5
PACCE	FOLFOX/BEV	397	13	7	0	1	NR
	FOLFOX/BEV/PAN	407	24	13	4	36	NR
PACCE	FOLFIRI/BEV	113	9	8	1	0	NR
	FOLFIRI/BEV/PAN	111	28	13	5	38	NR
CAIRO2	CAPOX/BEV	366	19.1	16.7	NR	20.8	4.1
	CAPOX/BEV/cetuximab	366	26	12.3	NR	39.1	4.9
EPIC	Irinotecan	650	16.2	11.6	0.4	0.5	0.8
	Irinotecan + cetuximab	648	28.8	11.7	3.3	8.2	1.4
BOND	Cetuximab	115	1.7	4.3	NR	5.2	3.5
	Irinotecan + cetuximab	212	21.2	7.1	NR	9.4	0
NCIC CO 17	BSC	274	NR	11	0	0.4	0
	BSC + cetuximab	288	NR	11.2	5.8	11.8	4.5
PANITUMUMAB	BSC	234	0	1	0	9 (all grades)	0
	BSC + panitumumab	239	1	3	3	90 (all grades)	0
MABEL	Irinotecan + cetuximab	1147	19.4	5.3	NR	13.3	12.7

a Only 20% of patients had serum magnesium measurement.

FOLFOX=oxaliplatin/infused 5-FU/LV; BEV=bevacizumab; FOLFIRI=irinotecan/infused 5-FU/LV; BSC=best supportive care; PAN=panitumumab; NR=not reported.

**Table 5:** *K-ras* mutational analysis in randomised studies evaluating EGFR antibodies

**Study**	**No. of patients evaluable for *K-ras* mutation/No. of patients in the ITT study population**	**Proportion of patients with *K-ras* mutations**	**Treatment by mutation status**	**Response rates (%)**	***P*-value**	**Median progression-free survival**	***P*-value**	**Median overall survival**	***P*-value**
*First line*									
Van Cutsem *et al* (2009)	540/1198 (45%)	35.6% mutant	**Wild type**						
CRYSTAL			FOLFIRI	43.2	0.0025	8.7 months	0.02	21.0 months	HR: 0.84 (95% CI: 0.64–1.11)
			FOLFIRI +cetuximab	59.3		9.9 months		24.9 months	
			**Mutant**						
			FOLFIRI	40.2	0.46	8.1 months	0.75	17.7 months	HR: 1.03 (95% CI: 0.74–1.44)
			FOLFIRI +cetuximab	36.2		7.6 months		17.5 months	
Bokemeyer *et al* (2009)	233/337 (69%)	42% mutant	**Wild type**						
OPUS			FOLFOX	37	0.011	7.2 months	0.0163	NR	NR
			FOLFOX +cetuximab	61		7.7 months		NR	
			**Mutant**						
			FOLFOX	49	0.106	8.6 months	0.0192	NR	NR
			FOLFOX +cetuximab	33		5.5 months		NR	
									
Hecht *et al* (2009)	865/1053 (82%)	40% mutant	**Wild type**						
PACCE			FOLFOX + bevacizumab	56	NR	11.5 months	HR: 1.36 (95% CI: 1.04–1.77)	24.5	0.045
			FOLFOX + bevacizumab + panitumumab	50		9.8 months		20.7	
			**Mutant**						
			FOLFOX + bevacizumab	44	NR	11.0 months		19.3	
			FOLFOX + bevacizumab + panitumumab	47		10.4 months		19.3	
Tol *et al* (2009)	528/736 (72%)	39.6% mutant	**Wild type**						
CAIRO 2			CAPOX + bevacizumab	50.0	0.06	10.6 months	0.030	22.4 months	0.64
			CAPOX + bevacizumab +cetuximab	61.4		10.5 months		21.8 months	
			**Mutant**						
			CAPOX + bevacizumab	59.2	0.03	12.5 months	0.003	24.9 months	0.03
			CAPOX + bevacizumab +cetuximab	45.9		8.1 months		17.2 months	
Hecht *et al* (2009)	865/1053 (82%)	40% mutant	**Wild type**						
PACCE			FOLFIRI + bevacizumab	48	NR	12.5 months	NR	19.8	NR
			FOLFIRI + bevacizumab + panitumumab	54		10.0 months		NE	
			**Mutant**						
			FOLFIRI + bevacizumab	38	NR	11.9 months		20.5 months	
			FOLFIRI + bevacizumab + panitumumab	30		8.3 months		17.8 months	
									
*Subsequent lines*									
Tejpar *et al* (2008)	148/157 (94%)	39% mutant	**Wild type**						
EVEREST			Irinotecan +cetuximab (standard dose)	30.4	0.396	5.7 months for all wild-type patients	0.014 (in favour of wild type in standard dose)	NR	NR
			Irinotecan +cetuximab (escalating dose)	41.9				NR	
			**Mutant**				<0.0001		
			Irinotecan +cetuximab (standard dose)	0	NR	2.7 months for all mutant patients	(in favour of wild type in escalating dose)	NR	NR
			Irinotecan +cetuximab (escalating dose)	0				NR	
									
Amado *et al* (2008)	427/463 (92%)	43% mutant	**Wild type**						
			Panitumumab	17	NR	12.3 weeks	<0.0001	8.1 months	NS
			BSC	0		7.3 weeks		7.6 months	
			**Mutant**						
			Panitumumab	0	NR	7.4 weeks	0.99	4.9 months	NS
			BSC	0		7.3 weeks		4.4 months	
Karapetis *et al* (2008)	394/572 (69%)	42.3% mutant	**Wild type**						
NCIC CO.17			Cetuximab	12.8	NR	3.7 months	<0.001	9.5 months	<0.00
			BSC	0		1.9 months		4.8 months	1
			**Mutant**						
			Cetuximab	1.2	NR	1.8 months	0.96	4.5 months	0.89
			BSC	0		1.8 months		4.6 months	

ITT=intension to treat; FOLFOX=oxaliplatin-infused 5-FU/LV; FOLFIRI=irinotecan-infused 5-FU/LV; BSC=best supportive care; NR=not reported; NS=not significant; NE=not estimable.

**Table 6:** Randomised trials of oxaliplatin-infused 5-FU/leucovorin *vs* oxaliplatin/capecitabine

**Study**	**Treatment arms**	**Number of patients**	**Objective response rates (%)**	**Median PFS/TTP (months)**	**Median overall survival (months)**	**Comments**
*First line*						
Porschen *et al* (2007) German AIO	FUFOX	234	54	8.0	18.8	Primary end point=PFS
	CAPOX	242	48	7.1	16.8	Non-inferiority margin for 95% CI <1.29.
						HR: 1.17; 95% CI: 0.96–1.43, therefore 1° end point not met
Diaz-Rubio *et al* (2007) Spanish TTD	FUOX	174	46	9.5	20.8	Primary end point=TTP
	CAPOX	174	37	8.9	18.1	Non-inferiority margin for 95% CI <1.27.
						HR: 1.18; 95% CI: 0.9–1.5, therefore 1° end point not met
Ducreux *et al* (2007) French	FOLFOX 6	150	46	9.3	20.5	Primary end point=best response rate
	CAPOX	156	42	8.8	19.9	Non-inferiority margin for 95% CI <15%.
						Difference in response rate=4.7% upper limit of 95% CI=14.4%, therefore 1° end point just met
Cassidy *et al* (2008) XELOX -1	FOLFOX 4	1017	39	8.5	19.6	Primary end point=PFS
	CAPOX	1017	37	7.9	19.8	Non-inferiority margin for 97.5% CI < 1.23.
						HR: 1.05; 97.5% CI: 0.94–1.18, therefore 1° end point met
						
*Second line*						
Rothenberg *et al* (2008) XELOX -2	FOLFOX 4	314	12.4	5.5	13.2	Primary end point=PFS
	CAPOX	313	15.3	5.1	12.7	Non-inferiority margin for 95% CI <1.30.
						HR: 1.03; 97.5% CI: 0.87–1.24, therefore 1° end point met

FUFOX, FUOX and FOLFOX=different dose schedules of oxaliplatin/infused 5-FU/LV; PFS=progression free survival; TTP=time to tumour progression; HR=hazard ratio; CI=confidence interval.

**Table 7:** Randomised studies evaluating combination *vs* sequential treatment in advanced colorectal cancer

**Study**	**Treatment arms**	**Number of patients**	**First line response rates**	***P*-value**	**Median progression-free survival from first line treatment (months)**	***P*-value**	**Median overall survival (months)**	***P*-value**
Seymour *et al* (2007a, 2007b) FOCUS	Strategy A 5-FU/LV → irinotecan	710	28% (5-FU/LV)		6.3 (5-FU/LV)		13.9	
	Strategy B 5-FU/LV → FOLFIRI or FOLFOX	356 (FOLFIRI) 356 (FOLFOX)	28% (5-FU/LV)	<0.001 (strategy C *vs* A or B)	6.3 (5-FU/LV)	<0.001 (strategy C *vs* A or B)	15.1	NS
	Strategy C FOLFIRI → FOLFOX FOLFOX → FOLFIRI	356 (FOLFIRI) 357 (FOLFOX)	49% (FOLFOX or FOLFIRI)		8.5 (FOLFOX or FOLFIRI)		15.9	
Koopman *et al* (2007) CAIRO	Strategy A capecitabine → irinotecan → CAPOX	410	20% (capecitabine)	<0.0001	5.8 (capecitabine)	0.0002	16.3	0.3281
	Strategy B CAPIRI → CAPOX	410	41% (CAPIRI or CAPOX)		7.8 (CAPIRI or CAPOX)		17.4	
Cunningham *et al* (2009) LIFE	Strategy A 5-FU/LV → irinotecan	363	29.8% (5-FU/LV)	<0.0001	5.9 (5-FU/LV)	<0.0001	15.2	0.155
	Strategy B FOLFOX → irintoecan	362	54.1% (FOLFOX)		7.9 (FOLFOX)		15.9	

LV=leucovorin, FOLFOX=oxaliplatin/infused 5-FU/LV, FOLFIRI=irinotecan/infused 5-FU/LV, CAPOX=capecitabine/oxaliplatin, CAPIRI=capecitabine/irinotecan, NR=not reported, NS=non significant.
